# Controlling Synchronization of Spiking Neuronal Networks by Harnessing Synaptic Plasticity

**DOI:** 10.3389/fncom.2019.00061

**Published:** 2019-09-04

**Authors:** Joseph Schmalz, Gautam Kumar

**Affiliations:** Department of Chemical and Materials Engineering, University of Idaho, Moscow, ID, United States

**Keywords:** synchronization, desynchronization, spiking neural network, spike-timing dependent plasticity, harnessing plasticity

## Abstract

Disrupting the pathological synchronous firing patterns of neurons with high frequency stimulation is a common treatment for Parkinsonian symptoms and epileptic seizures when pharmaceutical drugs fail. In this paper, our goal is to design a desynchronization strategy for large networks of spiking neurons such that the neuronal activity of the network remains in the desynchronized regime for a long period of time after the removal of the stimulation. We develop a novel “Forced Temporal-Spike Time Stimulation (FTSTS)” strategy that harnesses the spike-timing dependent plasticity to control the synchronization of neural activity in the network by forcing the neurons in the network to artificially fire in a specific temporal pattern. Our strategy modulates the synaptic strengths of selective synapses to achieve a desired synchrony of neural activity in the network. Our simulation results show that the FTSTS strategy can effectively synchronize or desynchronize neural activity in large spiking neuron networks and keep them in the desired state for a long period of time after the removal of the external stimulation. Using simulations, we demonstrate the robustness of our strategy in desynchronizing neural activity of networks against uncertainties in the designed stimulation pulses and network parameters. Additionally, we show in simulation, how our strategy could be incorporated within the existing desynchronization strategies to improve their overall efficacy in desynchronizing large networks. Our proposed strategy provides complete control over the synchronization of neurons in large networks and can be used to either synchronize or desynchronize neural activity based on specific applications. Moreover, it can be incorporated within other desynchronization strategies to improve the efficacy of existing therapies for numerous neurological and psychiatric disorders associated with pathological synchronization.

## 1. Introduction

Most of the existing therapies for neurological disorders focus on suppressing symptoms and ignore the dynamical aspects of the underlying network that create the pathological symptoms. Therapies that purely focus on suppressing the pathological symptoms instead of addressing aberrant dynamic characteristics of the network could fail to achieve a long term behavioral recovery (Popovych and Tass, 2014). One such example is the treatment of Parkinson's Disease motor symptoms by suppressing the strong oscillatory firing pattern of neurons in the brain using high frequency stimulation (HFS) (Temperli et al., 2003; Hammond et al., 2007; Benabid et al., 2009; Postuma et al., 2015; Singh, 2018). The applied HFS suppresses the oscillatory activity and the Parkinsonian motor symptoms while the stimulation is on but when the stimulation is off, the oscillations and motor symptoms reemerge (Temperli et al., 2003; Deuschl et al., 2006). Additionally, epilepsy is also characterized by an excessive synchronous firing pattern of neurons, which causes epileptic seizures (Duncan et al., 2006). Similar to Parkinson's diseases, HFS helps to control the excessive synchronization of neurons and epileptic seizures (Klinger and Mittal, 2016). To improve upon open-loop stimulation protocol, new neural stimulation strategies have been developed to more effectively and efficiently desynchronize pathologically synchronous neuronal networks throughout the brain.

Recently, control-theoretic approaches have been used to design more effective and energy efficient desynchronization strategies (Popovych and Tass, 2014). These optimal stimulation protocols include a single pulse minimum energy desynchronizing control input (Deuschl et al., 2006; Nabi et al., 2013a,b; Mauroy et al., 2014; Wilson and Moehlis, 2014; Monga et al., 2018) and closed-loop delayed feedback (Hauptmann et al., 2005; Popovych et al., 2005, 2017; Kiss et al., 2007; Vlachos et al., 2016) approaches. The minimum energy control input approach designs a pulse that pushes the state of the network to a phaseless set-point, which is the point where all the isochrons of the system converge (Nabi et al., 2013a,b). Here, the network's inherent noise randomly pushes each individual neuron onto a different isochron with its own phase. The net effect of this random reset is an asynchronous population activity. Another approach is closed-loop delayed feedback control. In the closed-loop delayed feedback control approach (Vlachos et al., 2016), the time-delayed average population activity is used as a feedback to desynchronize the network. Since this approach only feeds the past population activity, the applied desynchronizing input is only active whenever the network becomes synchronous. While these approaches provide an optimal desynchronization strategy, most of them assume that the network connections are static and ignore the inherent plastic nature of neuronal synapses (Abbott and Nelson, 2000).

Hebbian plasticity is a well-known form of activity-dependent synaptic plasticity (Abbott and Nelson, 2000). This form of plasticity enforces productive connections between neurons that produce action potentials and depresses unproductive connections that do not elicit action potentials (Hebb, 1949). One form of activity-dependent synaptic plasticity is spike-time dependent plasticity (STDP) (Song et al., 2000). This rule increases the weight of a synaptic connection when the pre-synaptic neuron fires before the post-synaptic neuron within a given time window and decreases the weight when the order is reversed (Song et al., 2000). An increase or decrease in the synaptic weight is coined long-term-potentiation (LTP) or long-term-depression (LTD), respectively. The introduction of plasticity into a neuronal network creates multiple stability points with different levels of synchronous activity (Tass and Hauptmann, 2007; Pfister and Tass, 2010; Popovych and Tass, 2014). Since the connections are plastic, an external stimulus can move the network from one stability point to another in order to drive the network from a synchronous to an asynchronous state.

To incorporate the synaptic plasticity within the desynchronization stimulation protocol, Coordinate Reset (CR) based stimulation strategies have been developed, which temporarily makes the pathologically synchronous stability point unstable to move the network into the asynchronous state by harnessing synaptic plasticity (Tass, 2003a,b; Tass and Majtanik, 2006; Tass and Hauptmann, 2007; Pfister and Tass, 2010; Ebert et al., 2014; Zeitler and Tass, 2015). In this approach, the network is driven to the asynchronous regime in the presence of external stimulation because of the inherent domination of LTD in asynchronous bistable networks. Once CR stimulation moves the network from the synchronous to the asynchronous stability point, the stimulation input is no longer required (Tass and Hauptmann, 2007; Pfister and Tass, 2010). This results in a long-lasting desynchronization of the network after the stimulation protocol is turned off (Tass and Majtanik, 2006). While this protocol is effective, it has its own limitations. CR based stimulation strategies rely on acute desynchronization of the networks regardless of the plasticity rules during the stimulation. If the network is not bistable, the acute-desynchronization of the network may not depress the synaptic weight of the network and the desynchronization of the network will be brief (Pfister and Tass, 2010). This lack of bistability may arise in a network where LTP dominates LTD (i.e., pathological networks). Moreover, this strategy only provides a way to desynchronize neural activity and not to control the synchronization level of the network.

In this work, we have developed a novel stimulation strategy “Forced Temporal Spike-Time Stimulation (FTSTS)” which addresses above shortcomings of the CR-based stimulation approach. While all other stimulation strategies focus on desynchronizing neural activity within a network, our strategy focuses on harnessing the underlying synaptic plasticity of the network to control the average network synaptic strength by forcing the spiking neurons to fire in specific temporal patterns. Thus, our strategy provides complete control over the synchrony level of networks for a long period of time, not just desynchronization. We demonstrate the efficacy of FTSTS strategy in controlling the desired synchrony level in large excitatory-inhibitory (E-I) networks. We show in simulation that the FTSTS strategy can effectively desynchronize the neural activity in networks where LTP dominates LTD on average. Further, we combine the FTSTS strategy with the CR stimulation strategy to demonstrate how this can enhance the overall performance of the CR stimulation strategy in desynchronizing large networks.

The paper begins with a description of models used to describe the spiking E-I networks dynamics, STDP rules, and a measure of synchrony as well as the stability analysis of E-I networks in section 2. In section 3, we first provide a mechanistic understanding of the FTSTS strategy by considering control of synchrony in a two neuron E-I network. We then demonstrate the efficacy of the FTSTS strategy in desynchronizing large E-I networks subject to different plasticity rules, and uncertainties in the network parameters and the designed stimulation parameters. Finally, we show how the FTSTS strategy can be incorporated within the CR stimulation strategy to improve the overall performance of the CR stimulation strategy. The paper ends with a detailed discussion on the comparison of our approach with the existing stimulation strategies for desynchronization of spiking neural networks as well as the limitations of our strategy in section 4.

## 2. System Model

### 2.1. Excitatory-Inhibitory (EI) Network Model

We consider networks of 2, 000 and 10, 000 spiking neurons consisting of 80% excitatory (E) and 20 % inhibitory (I) neurons (Brunel and Hansel, 2006; Vlachos et al., 2016). The following Leaky-Integrate-and-Fire (LIF) model describes a single excitatory or inhibitory neuron's dynamics in the E-I network.

(1) $$\begin{array}{rcl} \tau_{m}\frac{dv_{E}\left(t\right)}{dt} & = & -v_{E}\left(t\right)+Z_{E}\left(t\right)+\mu_{E}+\sigma_{E}\sqrt{\tau_{m}}\chi \left(t\right)+V_{stim}^{E}\left(t\right), \end{array}$$

(2) $$\begin{array}{rcl} \tau_{m}\frac{dv_{I}\left(t\right)}{dt} & = & -v_{I}\left(t\right)+Z_{I}\left(t\right)+\mu_{I}+\sigma_{I}\sqrt{\tau_{m}}\chi \left(t\right)+V_{stim}^{I}\left(t\right). \end{array}$$

Here, *v*_*E*_(*t*) and *v*_*I*_(*t*), in millivolts (mV), represent the membrane potential of the excitatory and inhibitory neurons, respectively. τ_*m*_ (in ms) is the membrane time constant. *Z*_*i*_(*t*) denotes the synaptic input to the *i*^*th*^ population of neurons where *i* ∈ {*E, I*}. The synapses between the excitatory and inhibitory populations are randomly connected with a probability of ϵ. The synaptic input function:

(3) $$\begin{array}{r} Z_{i}\left(t\right)=\frac{J_{ij}}{C_{ij}}S_{ij}\left(t\right) \end{array}$$

defines the input to the *i*^*th*^ neuron population. In Equation (3), *J*_*ij*_ represents the synaptic strength between a presynaptic neuron in population *j* and postsynaptic neuron in population *i*, in mV, where *i* ∈ {*E, I*} and *j* ∈ {*E, I*}. For example, the synaptic strength of a I-to-E synapse is *J*_*EI*_. *C*_*ij*_ = 0.3*N*_*tot*_ denotes a scaling factor where *N*_*tot*_ is the total number of neurons in the network. *S*_*ij*_(*t*) is the synaptic function. The Gaussian distributed baseline current to the *i*^*th*^ type neuron is denoted as:

(4) $$\begin{array}{r} \mu_{i}+\sigma_{i}\sqrt{\tau_{m}}\chi \left(t\right) \end{array}$$

with a mean baseline current of μ_*i*_ and a variance of $\sigma_{i}^{2}\tau_{m}$. χ(*t*) is white noise with a mean of 0 and a variance of 1. Finally, $V_{stim}^{i}\left(t\right)$ denotes the external stimulation input to the *i*^*th*^ neuron population.

The synaptic function *S*_*ij*_(*t*) is modeled as (Brunel and Hansel, 2006):

(5) $$\begin{array}{rcl} \tau_{d}\frac{dS_{ij}\left(t\right)}{dt} & = & -S_{ij}\left(t\right)+X_{ij}\left(t\right), \end{array}$$

(6) $$\begin{array}{rcl} \tau_{r}\frac{dX_{ij}\left(t\right)}{dt} & = & -X_{ij}\left(t\right)+W_{ij}\left(t\right)\delta \left(t-t_{pre}+t_{delay}\right). \end{array}$$

Here, *X*_*ij*_ describes the input to the *i*^*th*^ population of neurons from the *j*^*th*^ population of neurons. The time constants governing the decay and rise time are τ_*d*_ (in ms) and τ_*r*_ (in ms), respectively. Synaptic connections between the *i*^*th*^ and *j*^*th*^ neuron populations are randomly connected with a probability of ϵ. The weight of each synaptic connection is defined as *W*_*ij*_. Throughout the work, we assume that E-to-I connections (*W*_*IE*_(*t*)) are plastic and the I-to-E connections (*W*_*EI*_) are static except in section 3.9 and Figure 11 where we consider both connections to be plastic. Unless otherwise specified, we further assume no synaptic connectivity among neurons in excitatory or inhibitory populations. The Dirac-Delta function δ(*t* − *t*_*pre*_ + *t*_*delay*_) models the synaptic input to a postsynaptic neuron from a presynaptic neuron when the presynaptic neuron fires at time *t*_*pre*_ (in ms) with a synaptic delay of *t*_*delay*_ (in ms).

### 2.2. Spike-Timing Dependent Plasticity (STDP) Model

The coupling value of the plastic E-to-I synapse (*W*_*IE*_(*t*)) is governed by STDP (Song et al., 2000), which is defined as follows:

(7) $$\begin{array}{r} W_{IE}\left(t+\Delta t\right)=W_{IE}\left(t\right)+\Delta W_{IE}\left(t\right), \end{array}$$

where Δ*W*_*IE*_(*t*) is given as:

(8) $$\Delta W_{IE}(t)=\eta_{e}a_{LTP}A_{post}(t)\;if\;t_{pre}-t_{post}<0,$$

(9) $$\Delta W_{IE}(t)=\eta_{e}a_{LTD}A_{pre}(t)\;if\;t_{pre}-t_{post}>0.$$

Here, Δ*W*_*IE*_(*t*) defines the change in the synaptic weight determined by the spike-time of a presynaptic (*t*_*pre*_) and postsynaptic (*t*_*post*_) neuron. The rate at which the E-to-I synaptic coupling changes is governed by the learning rate η_*e*_. Additionally, the relative contribution of LTD and LTP to Δ*W*_*IE*_(*t*) is denoted by *a*_*LTP*_ and *a*_*LTD*_. (Song et al., 2000; Ebert et al., 2014). Since LTD generally dominates LTP, *a*_*LTD*_ is 10% larger than *a*_*LTP*_. *A*_*post*_(*t*) and *A*_*pre*_(*t*) are described by the following two exponential functions:

(10) $$\begin{array}{rcl} \tau_{LTP}\frac{dA_{post}}{dt} & = & -A_{post}+A_{0}\delta \left(t-t_{post}\right), \end{array}$$

(11) $$\begin{array}{rcl} \tau_{LTD}\frac{dA_{pre}}{dt} & = & -A_{pre}+A_{0}\delta \left(t-t_{pre}\right). \end{array}$$

Here, the size of the LTP and LTD time window is defined by the STDP time constants τ_*LTP*_ and τ_*LTD*_. In a similar fashion to *a*_*LTP*_ and *a*_*LTD*_, τ_*LTD*_ is set to be 10% greater than τ_*LTP*_. Upon the firing of a presynaptic or postsynaptic neuron, a small value *A*_0_ is added to the appropriate exponential STDP decay function. The E-to-I synaptic weight is defined as the coupling value *W*_*IE*_(*t*) multiplied by the synaptic strength *J*_*IE*_ (i.e., *J*_*IE*_*W*_*IE*_(*t*)).

### 2.3. Synchrony Measurement

We measure the synchrony level of the network by computing the Kuramoto order parameter (*R*(*t*)) based on the spike times of neurons in the excitatory population (Kuramoto, 1984; Daido, 1992; Tass, 2007; Ebert et al., 2014). The phase (ϕ_*k*_(*t*)) of the *k*^*th*^ neuron in the excitatory population is calculated using the following equation:

(12) $$\begin{array}{r} \phi_{k}\left(t\right)=\frac{2\pi \left(t_{k,i+1}-t\right)}{t_{k,i+1}-t_{k,i}}, \end{array}$$

where *t*_*k,i*_ is the *i*^*th*^ spike time for the *k*^*th*^ neuron. The Kuramoto order parameter (*R*(*t*)) and the average phase of the neurons (ψ(*t*)) are calculated using:

(13) $$\begin{array}{r} R\left(t\right)e^{i\psi \left(t\right)}=\frac{1}{N_{E}}\overset{N_{E}}{\underset{k=1}{\sum }}e^{i\phi_{k}\left(t\right)}. \end{array}$$

Here, *N*_*E*_ represents the number of excitatory neurons. A highly synchronous network has a Kuramoto order parameter of *R*(*t*) = 1 and a completely asynchronous network has a value of *R*(*t*) = 0.

### 2.4. Determination of Synchronous and Asynchronous Regimes

**Figure 1:**
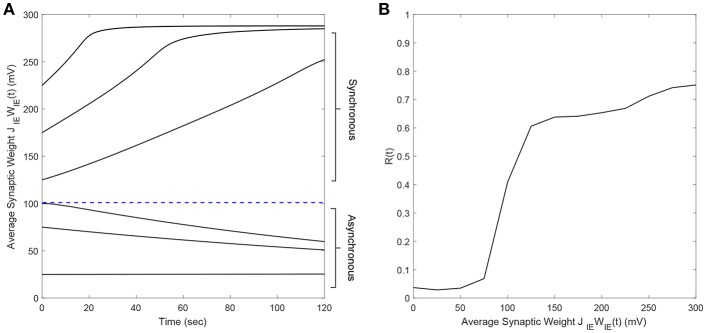
The synchrony level and stability points of a plastic 2,000 spiking neuron E-I network. **(A)** The average synaptic weight either converges to the maximum or minimum value. Each line represents the trajectory of the synaptic weight with a different initial condition. The stability threshold is depicted as a blue dashed line. **(B)** The synchrony level of the network, represented by the Kuramoto order parameter R(t), increases with increasing average synaptic weight [*J*_*IE*_*W*_*IE*_(*t*)].

It is well-known that plastic neural networks exhibit multiple stability points (Song et al., 2000; Tass and Hauptmann, 2007; Pfister and Tass, 2010; Popovych and Tass, 2014). Similar to other networks, our E-I network exhibits two stability points at a high and low average E-to-I synaptic weight value. Figure 1A shows the average E-to-I synaptic weight converging to either *J*_*IE*_*W*_*IE*_ = 10 mV or *J*_*IE*_*W*_*IE*_ = 290 mV. The average synaptic weight converges to *J*_*IE*_*W*_*IE*_ = 290 mV if the initial average synaptic weight is >100 mV and converges to *J*_*IE*_*W*_*IE*_ = 10 mV when it <100 mV. Additionally, we find that the network becomes more synchronous as the average E-to-I synaptic weight increases, which is shown in Figure 1B in terms of the Kuramoto order parameter *R*(*t*). Therefore, the network exhibits a high level of synchrony at high synaptic weights and a low level of synchrony at low synaptic weights.

### 2.5. Model Parameters

We use the model parameters defined in Table 1 unless stated otherwise. All the simulation are performed in Matlab R2016b. The differential equations are solved using Euler's method with a step size of 0.1 ms. All our codes and relevant simulation parameters to replicate the presented results in this manuscript are available on our research webpage (https://webpages.uidaho.edu/gkumar) and in the Supplementary Material.

**Table 1:** The model parameters of our E-I network.

**Neuron parameters**	**Value**	**Plasticity parameters**	**Value**
**Brunel and Hansel (2006); Vlachos et al. (2016)**		**Song et al. (2000); Hauptmann and Tass (2009)**	
*v*_*reset*_	0 mV	*W*_*EI*, 0_	1
*v*_*threshold*_	20 mV	ϵ	0.1
μ_*E*_	20.8 mV	*a*_*LTD*_	−1.1
μ_*I*_	18 mV	*a*_*LTP*_	1
σ_*E*_	1 mV	τ_*LTD*_	22 ms
σ_*I*_	3 mV	τ_*LTP*_	20 ms
τ_*m*_	10 ms	*A*_0_	0.005
τ_*d*_	1 ms	η_*e*_	0.25
τ_*r*_	1 ms	*c*_*P*_	0.038
τ_*delay*_	5 ms	*c*_*D*_	0.02
*J*_*EI*_	100 mV	τ_*P*_	10 ms
*J*_*IE*_*W*_*IE*_(*t*)	∈ [10, 290] mV	τ_*D*_	25 ms

## 3. Results

We begin by providing an insight into the underlying mechanism of our stimulation strategy “Forced Temporal Spike-Time Stimulation” (FTSTS) using an illustrative example of a two neuron E-I network in section 3.1. Next, we demonstrate the efficacy of the FTSTS strategy in controlling synchronized activity of a 2,000 and 10,000 neuron E-I networks (see sections 3.2, 3.3). We then show the robustness of the FTSTS strategy in the presence of uncertainties in the designed stimulation pulses, model parameters and network connectivity (see sections 3.4, 3.5, 3.6, 3.7). Next, in section 3.8, we combine the FTSTS strategy with the existing coordinate reset (CR) stimulation strategy to show the efficacy of the FTSTS-CR strategy over the CR stimulation strategy. Finally, in sections 3.9 and 3.10, we demonstrate that FTSTS strategy can desynchronize E-I networks with additional plastic synapses and symmetric spike-time plasticity rules.

### 3.1. Control of E-to-I Synaptic Weight in a Two Neuron Network

**Figure 2:**
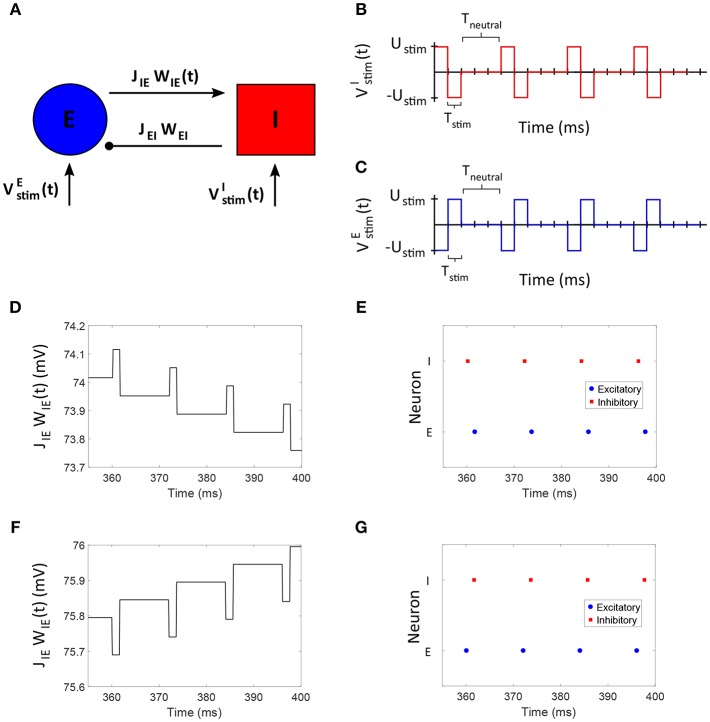
The FTSTS protocol for a two neuron E-I network. **(A)** Shows an excitatory-inhibitory network. **(B,C)** Show the FTSTS input pattern for the inhibitory and excitatory neuron, respectively. The FTSTS pulse parameters are *U*_*stim*_ = 300 mV, *T*_*stim*_ = 1 ms, and *T*_*neutral*_ = 10 ms. This FTSTS protocol depresses the E-to-I synaptic weight as shown in **(D)**. The drop in synaptic weight is due to FTSTS inducing a post-before-pre spiking pattern in the E-I network, which is shown in **(E)**. On the other hand, **(F)** Shows how swapping the FTSTS inputs to the excitatory and inhibitory neuron increases the E-to-I synaptic weight. This induces the pre-before-post spiking pattern shown in **(G)**.

We considered an excitatory-inhibitory (E-I) network of two neurons to develop our “Forced Temporal Spike-Time Stimulation” (FTSTS) strategy (Figure 2A). We set a scaling factor of *C*_*ij*_ = 10*N*_*i*_ and probability of connectivity of ϵ = 1 (see section 2.1 for the meaning of these variables). Based on the STDP rule of activity-dependent plasticity, we designed stimulation inputs for both the inhibitory ($V_{stim}^{I}\left(t\right)$) and excitatory ($V_{stim}^{E}\left(t\right)$) neuron that forced the postsynaptic inhibitory neuron to spike before the presynaptic excitatory neuron, as shown in Figures 2B,C, respectively. The protocol stimulated the postsynaptic inhibitory neuron and the presynaptic excitatory neuron using charge-balanced rectangular pulses with an equal and opposite amplitude (*U*_*stim*_). Figure 2E shows the induced firing patterns in neurons, which decreased the average E-to-I synaptic weight of the network as shown in Figure 2D. On the other hand, the average E-to-I synaptic weight increased when $V_{stim}^{E}\left(t\right)$ and $V_{stim}^{I}\left(t\right)$ were switched such that the presynaptic excitatory neuron fired before the postsynaptic inhibitory neuron. The increased synaptic weight observed from the induced spiking pattern in Figure 2G is shown in Figure 2F.

### 3.2. FTSTS Effectively Controls the Neuronal Synchronization in a 2000 Neuron Network

**Figure 3:**
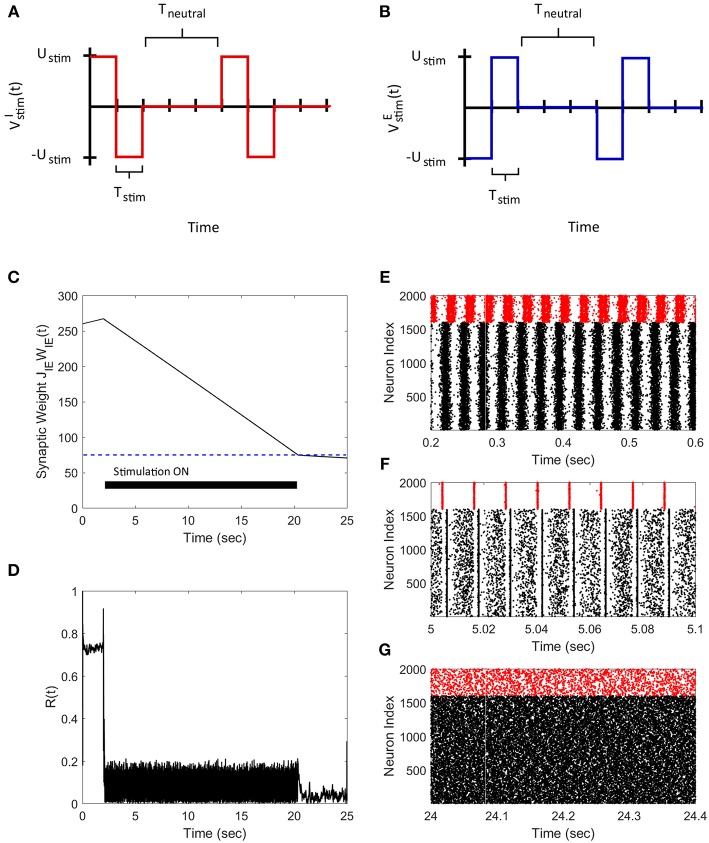
Desynchronization of neural activity in 2,000 neuron E-I network. **(A)** Shows the FTSTS waveform for inhibitory neurons. **(B)** Shows the FTSTS waveform for excitatory neurons. **(C)** Shows the time evolution of the average E-to-I synaptic weight. As shown here, the average E-to-I synaptic weight of network is decreased to 75 mV (blue-line), where the stimulation is turned off. **(D)** Shows the synchrony level of excitatory neurons as a function of time. **(E–G)** Show the spiking patterns before, during, and after the FTSTS protocol, respectively. The FTSTS pulse parameters are *U*_*stim*_ = 100 mV, *T*_*stim*_ = 1 ms, and *T*_*neutral*_ = 10 ms.

We applied the FTSTS strategy to a E-I network of 1,600 excitatory and 400 inhibitory neurons to demonstrate how our strategy can be used to control the synchrony of neuronal activity in large networks. In a larger network of neurons, our strategy forces the postsynaptic inhibitory population of neurons to spike before the presynaptic excitatory neuron population. We assumed that all the neurons in each specific population receives the same input. The applied FTSTS inputs to each neuron population are shown in Figures 3A,B. These inputs induced a specific spiking pattern, as shown in Figure 3F, which depressed the average E-to-I synaptic weight (shown in Figure 3C). The period of stimulation is highlighted with a solid black line in Figure 3C. Since the network has an asynchronous regime that converges to a low average E-to-I synaptic weight, we only required enough input to drive the network into the asynchronous regime. Therefore, we provided enough input to depress the synaptic weight of the network to 75 mV, which is slightly over the synchronous-asynchronous regime boundary (see Figure 1). As a result, the system naturally converged to the low synaptic weight stability point when the FTSTS protocol was turned off. The synchronous and asynchronous firing patterns before and after the stimulation protocol are displayed in Figures 3E,G, respectively. The synchrony level of the network as it transitioned from the synchronous to asynchronous regime is shown in Figure 3D. The activity of the E-I network prior to the applied stimulation (the first 2 s) was measured around *R*(*t*) = 0.75. When the stimulation protocol was turned on, the measured synchrony level became low (see Figure 3D). This is due to the asynchronous firing between each FTSTS pulse. When the FTSTS protocol was turned off, the network remained in the asynchronous regime at the measured network synchrony level of *R*(*t*) = 0.05 (see Figure 3D).

**Figure 4:**
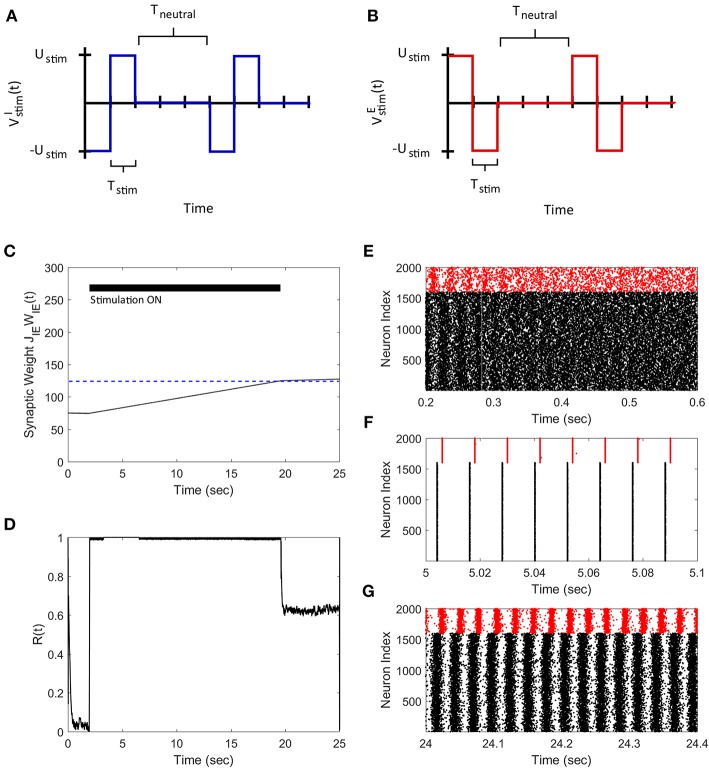
Resynchronization of neural activity in 2,000 neuron E-I network. **(A)** Shows the FTSTS waveform for inhibitory neurons. **(B)** Shows the FTSTS waveform for excitatory neurons. Note that the FTSTS waveforms for inhibitory and excitatory populations are swapped from the desynchronization case (see Figures 3A,B). **(C)** Shows the time evolution of the average E-to-I synaptic weight. As shown here, the average E-to-I synaptic weight of network is increased to 125 mV (blue-line), where the stimulation is turned off. **(D)** Shows the synchrony level of the network as a function of time. **(E–G)** Show the spiking patterns before, during, and after the FTSTS protocol, respectively. The FTSTS pulse parameters are *U*_*stim*_ = 200 mV, *T*_*stim*_ = 1 ms, and *T*_*neutral*_ = 10 ms.

Next, we demonstrate how our FTSTS strategy can also be used to synchronize the asynchronous network activity. To do so, we swapped the inputs to the inhibitory and excitatory neurons used in the desynchronization case, which are shown in Figures 4A,B. This stimulation protocol forced the presynaptic excitatory neuron population to fire prior to the postsynaptic inhibitory neuron populations, which is shown in Figure 4F. Similar to the two neuron case, swapping the inputs to the excitatory and inhibitory neurons induced LTP in the network and increased the average E-to-I synaptic weight of the network, as shown in Figure 4C. Again we are only required to drive the network into the synchronous regime (i.e., the average E-I synaptic weight above 100 mV) to synchronize the network. Therefore, the stimulation protocol was turned off when the average E-to-I synaptic weight reached 125 mV, which was slightly over the threshold. Here, the network will remain in the synchronous regime and the average E-to-I synaptic weight will converge to the high synaptic weight stability point. The spiking patterns of the E-I network before, during, and after the FTSTS protocol are shown in Figures 4E–G, respectively. Figure 4D shows the changes in the network synchrony level before, during, and after the FTSTS protocol. As shown in Figure 4D, the network synchrony increased from *R*(*t*) = 0.05 to *R*(*t*) = 0.6 after the removal of the stimulation. It should be noted that the synchrony level increases significantly during the stimulation in this case compared to the case of desynchronization (see Figure 3D for comparison). This is due to the large input of *U*_*stim*_ = 200 mV compared to the input used in Figure 3, which forces the neurons to fire in a highly synchronous firing pattern. If a smaller input is used to resynchronize the network, it would be more noisy during the FTSTS protocol and the FTSTS would be required for a longer period of time to resynchronize the network.

### 3.3. Desynchronization of Neural Activity in a Large E-I Network

**Figure 5:**
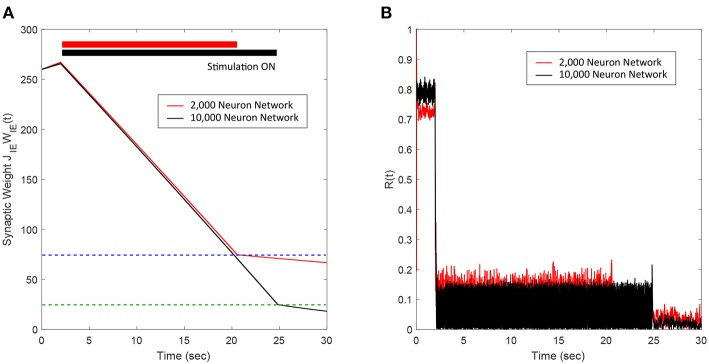
Desynchronization of neural activity in 10,000 neuron E-I network. **(A)** Shows the decrease in the average E-to-I synaptic weight of the network during and after the FTSTS stimulation (black line), which is compared to the decrease of the average synaptic of the 2,000 neuron E-I network (red line) by FTSTS. Due to the change in network dynamics for the 10,000 neuron network, the synaptic weight was decreased to 25 mV in order to push it into the asynchronous regime (green-dashed line). **(B)** Shows the network synchrony level during and after the FTSTS stimulation. The FTSTS pulse parameters used in this simulation are *U*_*stim*_ = 100 mV, *T*_*stim*_ = 1 ms, and *T*_*neutral*_ = 10 ms.

We applied our FTSTS protocol to demonstrate its applicability in larger networks. For demonstration purpose, we considered a E-I network with 8,000 excitatory and 2,000 inhibitory neurons. We set the probability of connectivity of the E-to-I and I-to-E synapses ϵ to 0.01. The FTSTS protocol induced the same post-before-pre firing patterns in the larger network which decreased the average E-to-I synaptic weight, as shown in Figure 5A. The stimulation protocol desynchronized the network in ~22 s, which is comparable to the desynchronization time of the 2,000 neuron network. The changes in the network synchrony level before, during, and after the FTSTS protocol are shown in Figure 5B. As noted in Figure 5B, the initial synchrony level of *R*(*t*) = 0.8 is reduced to approximately *R*(*t*) = 0.05 after the FTSTS protocol. Once the stimulation protocol reduced the average E-to-I synaptic weight below 75 mV (i.e., the asynchronous regime), we no longer required the external inputs to keep the network in the asynchronous regime.

### 3.4. Robustness to Uncertainties in the FTSTS Pulse Parameters

**Figure 6:**
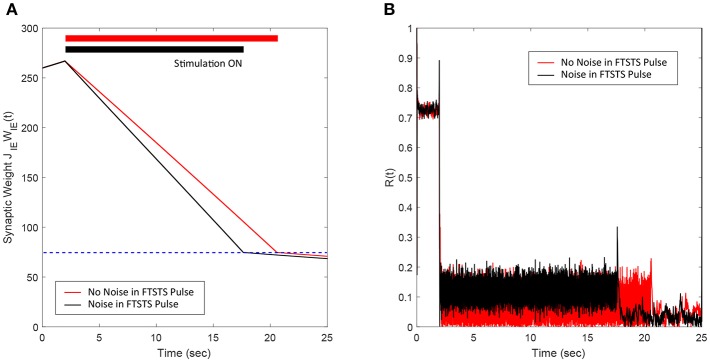
Robustness of the FTSTS strategy against random variations in the FTSTS pulse amplitude. The FTSTS pulse amplitude for each pulse has been chosen from a Gaussian distribution with mean *U*_*stim*_ and a variance of $\frac{U_{stim}}{10}$. **(A)** Shows the decrease in the average E-to-I synaptic weight of the network during and after the FTSTS stimulation (black line). The red line shows the decrease in average synaptic weight of a network without random variation in the applied FTSTS pulse amplitude. **(B)** Shows the network synchrony level during and after the FTSTS stimulation. The FTSTS pulse parameters used in this simulation are *U*_*stim*_ = 100 mV, *T*_*stim*_ = 1 ms, and *T*_*neutral*_ = 10 ms.

Here, we demonstrate the robustness of our protocol in desynchronizing a 2,000 neuron E-I network against uncertainties in the FTSTS pulse parameters. In particular, we considered uncertainty in the FTSTS pulse amplitude *U*_*stim*_, which we modeled in the form of a Gaussian distribution with mean *U*_*stim*_ and variance $\frac{U_{stim}}{10}$. Each of the applied pulse amplitude during stimulation was randomly chosen from this distribution. As shown in Figure 6B, the FTSTS strategy efficiently desynchronized the network by driving the network into the asynchronous regime. Figure 6A shows the changes in the average synaptic weight of the network before, during, and after the FTSTS protocol.

### 3.5. Robustness to Uncertainties in the Network Model Parameters

**Figure 7:**
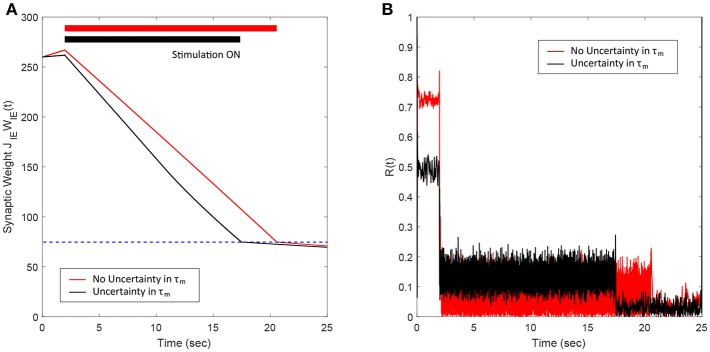
Robustness of the FTSTS strategy against uncertainty in the membrane time constant of neurons in the 2,000 neuron E-I network. **(A)** Shows the changes in the average E-to-I synaptic weight of the network, which is compared to a network without uncertainty in the membrane time constant (red line). **(B)** Shows the network synchrony level of the network throughout the simulation where the membrane time constant τ_*m*_ of individual neurons in the network is drawn from a uniform distribution U(8,12). The applied FTSTS pulse parameters are *U*_*stim*_ = 100 mV, *T*_*stim*_ = 1 ms, and *T*_*neutral*_ = 10 ms.

In this section, we show the robustness of our FTSTS strategy against uncertainties in the network model parameters. For demonstration, we considered variations in the membrane time constant τ_*m*_ of neurons in the network. We randomly assigned the membrane time constant τ_*m*_ of individual neurons in the 2,000 neuron E-I network from a uniform distribution U(8,12) to show the efficacy of our FTSTS strategy in desynchronizing the network activity. Figure 7A shows our simulation results for $\tau_{m}\in U\left(8,12\right)$. Here, the FTSTS protocol forced the average E-to-I synaptic weight of the network into the asynchronous regime within ~15 s of stimulation which led to desynchronization of the network activity after the removal of the stimulation, as shown in Figure 7B. As noticed here, the stimulation protocol desynchronized the network faster in this case compared to the case in Figure 3C where there is no variation in the membrane time constant. This is not surprising as an increase variability in the membrane time constant would induce more noise and desynchronize the firing pattern of the neurons initially, which is seen in Figure 7B with a lower initial Kuramoto order parameter value.

### 3.6. Addition of E-to-E and I-to-I Synaptic Connections

**Figure 8:**
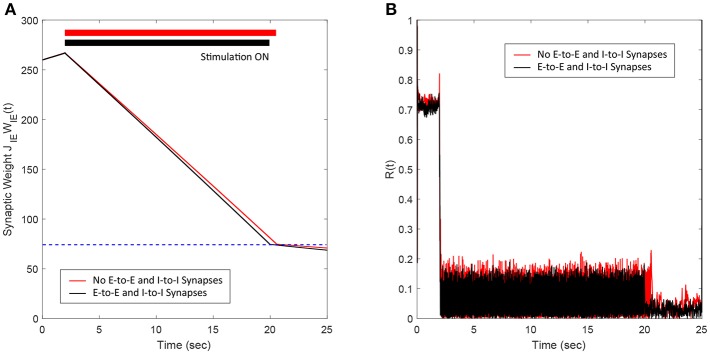
Efficacy of the FTSTS strategy in desynchronizing 2,000 neuron E-I network in the presence of E-to-E and I-to-I synaptic connectivity (black line). **(A,B)** Show the changes in the average E-to-I synaptic weight of the network and the network synchrony level, respectively. The red line in **(A)** Shows the decrease in average synaptic weight of a network with only E-to-I and I-to-E synapses. The FTSTS pulse parameters are *U*_*stim*_ = 100 mV, *T*_*stim*_ = 1 ms, and *T*_*neutral*_ = 10 ms.

In this section, we show the efficacy of the FTSTS strategy in desynchronizing 2000 neuron E-I network in the presence of E-to-E and I-to-I synaptic connectivity. We assumed that the synaptic strength of all synapses within the network are static except E-to-I synapses. We set the synaptic strength of the static I-to-E, E-to-E and I-to-I synapses as *J*_*EI*_ = 90 mV, *J*_*EE*_ = 50 mV, and *J*_*II*_ = 50 mV, respectively, with scaling factors of *C*_*EE*_ = *N*_*tot*_ and *C*_*II*_ = *N*_*tot*_, where *N*_*tot*_ = *N*_*E*_+*N*_*I*_. The probability of connectivity of the E-to-E and I-to-I was 0.1. The addition of E-to-E and I-to-I synapses within the E-I network didn't change the bifurcation of the regime into synchronous and asynchronous with respect to the network average E-I synaptic weight qualitatively. Our simulation results show that the FTSTS strategy effectively desynchronized the network activity, shown in Figure 8B, in the presence of E-to-E and I-to-I synapses by driving the average E-to-I synaptic weight of the network into the asynchronous stability regime, as shown in Figure 8A.

### 3.7. Robustness to Partial Spatially Inseparable Excitatory and Inhibitory Neuron Population

**Figure 9:**
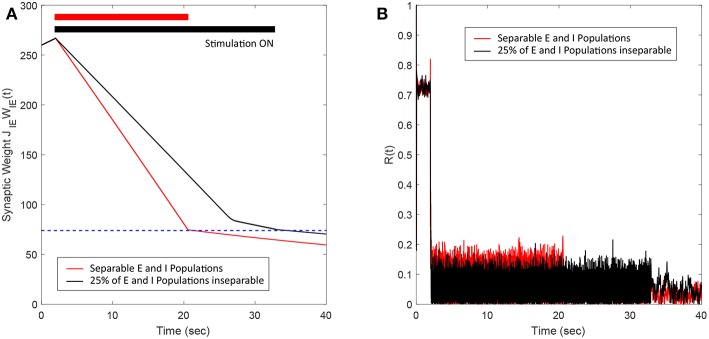
Efficacy of the FTSTS strategy in desynchronizing 2,000 neuron E-I network where 25% of the population is inseparable and receives both the excitatory and inhibitory population input. **(A)** Shows the change in the average E-to-I and I-to-E synaptic weight of the network (black line), which is compared to the completely separable case considered in Figure 3 (red line). **(B)** Shows the network synchrony level during the simulation. The FTSTS pulse parameters are *U*_*stim*_ = 100 mV, *T*_*stim*_ = 1 ms, and *T*_*neutral*_ = 10 ms.

In this section, we show the robustness of the FTSTS strategy for a case where E-I populations are not well separated. We assume that 25% of the excitatory and 25% of the inhibitory population are not spatially separable. Therefore, this inseparable population of E-I neurons receives inputs designed for the excitatory and the inhibitory population. Figure 9A shows that the FTSTS strategy is still able to push the average synaptic weight of the network into the asynchronous regime. The change in slope during the FTSTS protocol in Figure 9A is most likely due to the synaptic weight of the separable populations reaching the minimum weight value. At this point, the disruption from the stimulation protocol and the low average synaptic weight of the network helped to further depress the synaptic weight of the neurons. Figure 9B shows the reduction of the network synchrony with the Kuramoto order parameter dropping from *R*(*t*) = 0.72 to *R*(*t*) = 0.05.

### 3.8. Integration of FTSTS With the Coordinate Reset Strategy

**Figure 10:**
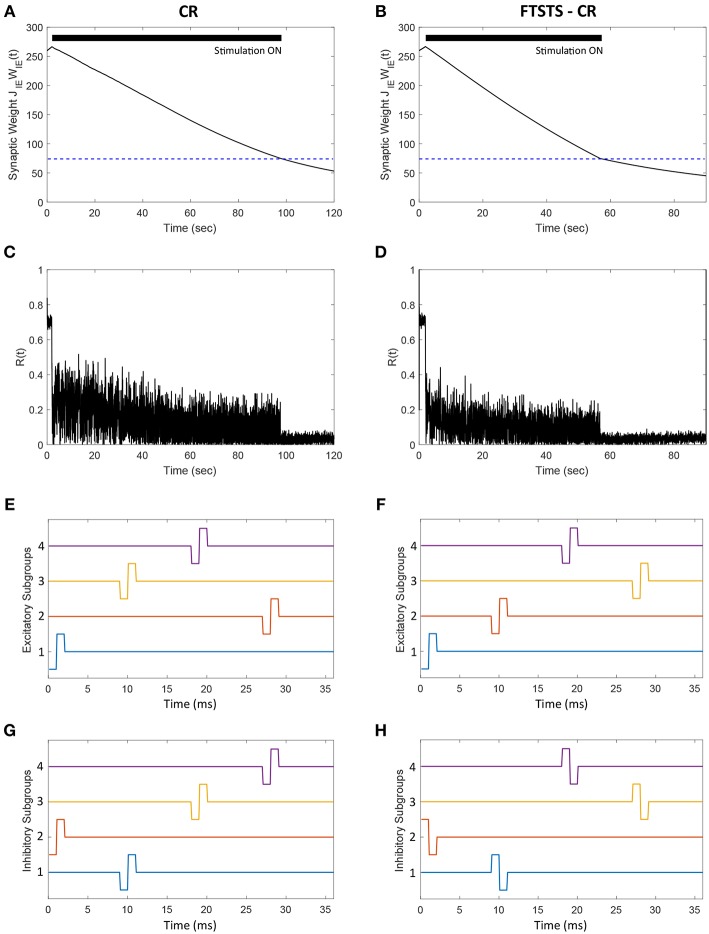
Efficacy of the FTSTS-CR strategy in desynchronizing 2,000 neuron E-I network in the presence of E-to-E and I-to-I synaptic connectivity. Each excitatory and inhibitory population of neurons is individually divided into 4 subpopulations (8 subpopulations for the E-I network). **(A,C)** Show the changes in the average E-to-I synaptic weight of the network and the network synchrony level, respectively, for the CR desynchronization strategy. The FTSTS-CR is compared to the CR approach in **(B,D)**, which show the changes in the average E-to-I synaptic weight of the network and the network synchrony level, respectively. **(E,G)** Show one cycle of coordinate reset (CR) stimulation applied to the subpopulations in each excitatory and inhibitory neuron population. **(F,H)** Show one cycle of the FTSTS-CR stimulation protocol applied to the excitatory and inhibitory populations. The designed FTSTS-CR and CR pulse parameters are *U*_*stim*_ = 100 mV, *T*_*stim*_ = 1 ms, and *T*_*neutral*_ = 7 ms.

In this section, we demonstrate how our FTSTS strategy could be incorporated within the standard coordinate reset (CR) stimulation protocol to effectively stimulate a large population of neurons. One way to implement the CR stimulation protocol is to divide the synchronous population of neurons into four subpopulations, which receive separate but identical inputs at different times over the course of period T (Tass, 2003a,b). T is the overall period of the synchronous neuron population without input. If the neuron population is divided into four subpopulations, then each subpopulations approximately will receive input every *T*/4. The order that each subpopulation receives input is randomly assigned at every period. In order to compare the CR to the FTSTS strategy, we individually divide the excitatory and inhibitory population into four subpopulations (8 subpopulations for the E-I network). Figures 10E,G show the CR stimulation pattern applied for one period to the excitatory and inhibitory population, respectively. These figures show the stimuli provided to the first quarter of neurons in the E-I network with one pulse to excitatory subpopulation 1 (Figure 10E, blue) and one pulse to the inhibitory subpopulation 2 (Figure 10G, orange). The stimuli to the second quarter of the E-I network is a pulse delivered to the excitatory subpopulation 3 (Figure 10E, yellow) and a pulse to the inhibitory subpopulation 1 (Figure 10G, blue). This is repeated for the remaining subpopulations in the excitatory and inhibitory populations. After every subpopulation has been stimulated over period T, a new random stimulation order is assigned for each subpopulation. The efficiency of the CR approach is shown in Figure 10A. Here, the CR stimulation depresses the average synaptic weight of the network over the course of ~100 s. This causes a drop in synchrony from *R*(*t*) = 0.7 to *R*(*t*) = 0.05, which is shown in Figure 10C.

We integrated this strategy with our FTSTS strategy and applied it to an E-I network, consisting of 2,000 neurons, in the presence of E-to-E and I-to-I synaptic connectivity. We randomly divided each excitatory and inhibitory population into four subpopulations. Then, we adjusted the CR stimulation pattern to incorporate our FTSTS protocol (FTSTS-CR), such that each randomly selected pair of excitatory and inhibitory subpopulations are forced to spike post-before-pre. The FTSTS-CR stimulation pattern for one period is shown in Figures 10F,H. For a single FTSTS-CR pulse, we set *U*_*stim*_ = 100 mV, *T*_*stim*_ = 1 ms and *T*_*neutral*_ = 7 ms. Similar to CR, we repeated this stimulation protocol for the other subpopulations in a random sequence for one period. Then, a new stimulation order is assigned for the next period T. Our protocol forces a randomly selected inhibitory subpopulation to fire prior to a randomly selected excitatory subpopulation, which over the course of 60 s depresses the average E-to-I synaptic weight of the entire population into the asynchronous regime as shown in Figure 10B. When the average E-to-I synaptic weight reached a preset value of 75 mV (asynchronous regime), we turned off the stimulation protocol. Figure 10D shows that the network remained in the desynchronized state at a synchrony level of *R*(*t*) = 0.05 for the rest of the simulation after removal of the FTSTS-CR protocol.

### 3.9. Robustness to Additional Plastic Synapses

**Figure 11:**
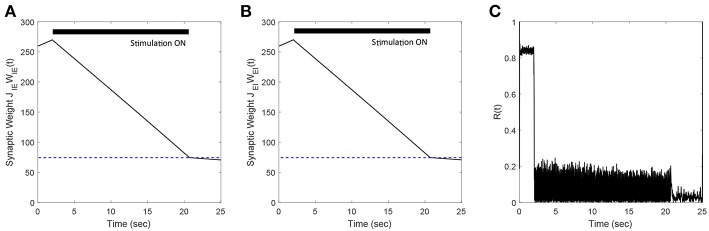
Efficacy of the FTSTS strategy in desynchronizing 2,000 neuron E-I network in the presence of Hebbian E-to-I and anti-Hebbian I-to-E plasticity. **(A,B)** Show the change in the average E-to-I and I-to-E synaptic weight of the network, respectively. **(C)** Shows the network synchrony level during the simulation. The FTSTS pulse parameters are *U*_*stim*_ = 100 mV, *T*_*stim*_ = 1 ms, and *T*_*neutral*_ = 10 ms.

In previous sections, we presented our results for networks where we considered only E-to-I plastic synapses. In this section, we demonstrate the efficacy of our FTSTS strategy in desynchronizing networks where both E-to-I and I-to-E synaptic connections are plastic. We modeled the plasticity dynamics of I-to-E synapses using an anti-Hebbian STDP plasticity rule (Bell et al., 1997; Luz and Shamir, 2012). For anti-Hebbian STDP, we used (Equations 8, 9) with the changed parameters *a*_*LTD*_ = 1 and *a*_*LTP*_ = −1.1 so that pre-before-post spike times decrease and post-before-pre spike times increases the synaptic weight. Our simulation results (see Figures 11A,B) show that the FTSTS strategy can potentially desynchronize the network by depressing both the E-to-I and I-to-E synaptic weights. Additionally, Figure 11C shows a decrease in the network synchrony from *R*(*t*) = 0.82 to *R*(*t*) = 0.05.

### 3.10. Robustness to a Symmetric Plasticity Rule

We demonstrate how a modified FTSTS protocol is able to control the synaptic weight of an E-I network with a symmetric plasticity rule. We use the same network described in section 2 with the following changes to the plasticity rule. The E-to-I synaptic weight is govern by the following equation (Hauptmann and Tass, 2009; Tass and Hauptmann, 2009):

(14) $$\begin{array}{r} \Delta W_{IE}\left(t\right)=c_{P}e^{-|ISI_{IE}|/\tau_{p}}-c_{D}e^{-|ISI_{IE}|/\tau_{D}}, \end{array}$$

where *ISI*_*IE*_ is the inter-spike-interval between spike-times of inhibitory and excitatory neurons in an E-to-I synapse. *c*_*P*_ and *c*_*D*_ are the potentiation and depotentiation learning rates, respectively. τ_*P*_ is the potentiation time constant and τ_*D*_ is the depotentiation time constant. The symmetric plasticity parameters can be found in Table 1.

**Figure 12:**
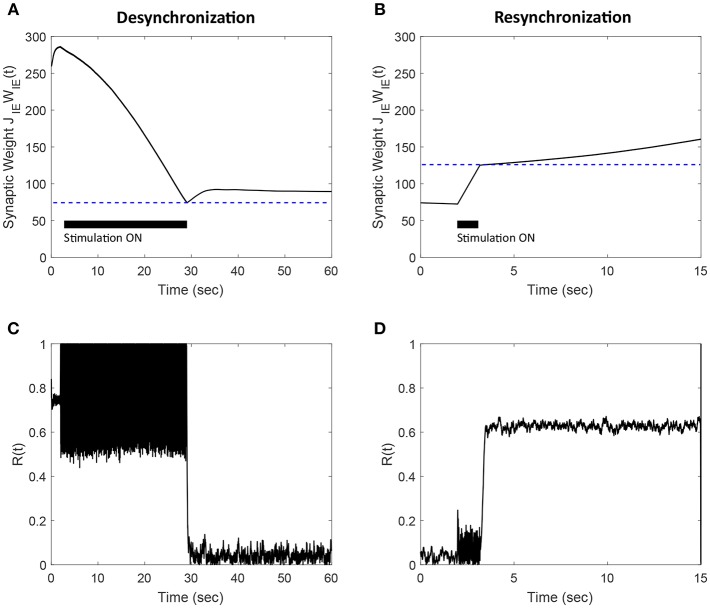
Efficacy of the FTSTS strategy in desynchronizing **(A,C)** and resynchronizing **(B,D)** a 2,000 neuron E-I network with symmetric plasticity. **(A)** Shows the decrease in average synaptic weight of the network with the modified FTSTS protocol for symmetric plasticity. **(C)** shows the change in the synchrony of the network before, during, and after stimulation. **(B)** Shows the increase in average synaptic weight of the network with the modified FTSTS protocol for symmetric plasticity. **(D)** Shows the change in the synchrony of the network before, during, and after stimulation. The FTSTS parameters used to decrease the average E-to-I synaptic weight are *U*_*stim*_ = 200 mV, *T*_*stim*_ = 1 ms, *T*_*neutral*_ = 22 ms, and *T*_*offset*_ = 11 ms. The FTSTS parameters used to increase the average E-to-I synaptic weight are *U*_*stim*_ = 100 mV, *T*_*stim*_ = 1 ms, *T*_*neutral*_ = 10 ms, and *T*_*offset*_ = 5 ms.

In order to apply our FTSTS protocol for desynchronizing E-to-I network with a symmetric plasticity rule, we modified the FTSTS protocol by offsetting the pulse to the inhibitory population by *T*_*offset*_. This forced ISIs that promote either an increase or decrease in the synaptic weight. Using our modified FTSTS protocol, we show in Figures 12A,C that our FTSTS strategy can efficiently depress the synaptic weight of the synchronous network and desynchronize the network by forcing larger *ISI*_*IE*_ values. Additionally, our protocol can also increase the average synaptic weight of the network to resynchronize the network by forcing short *ISI*_*IE*_ values. Figure 12B shows the increase in the average E-to-I synaptic weight. The subsequent increase in the synchrony level is shown in Figure 12D, where the order parameter increases from *R*(*t*) = 0.05 to *R*(*t*) = 0.6.

## 4. Discussions

**Figure 13:**
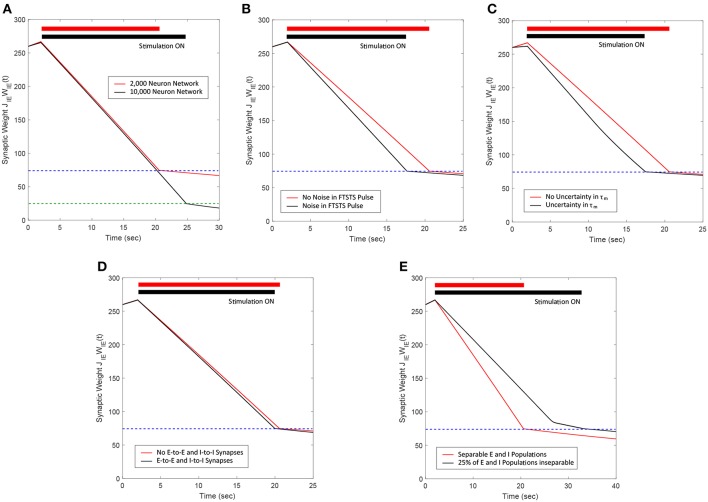
Summary of the robustness studies (red line) of the FTSTS approach with varying network and input parameters compared to the base E-I network in Figure 3 (black line). **(A)** Desynchronization of neural activity in 10,000 neuron E-I network. **(B)** Robustness of the FTSTS strategy against random variations in the FTSTS pulse amplitude. **(C)** Robustness of the FTSTS strategy against uncertainty in the membrane time constant of neurons in the 2,000 neuron E-I network. **(D)** Efficacy of the FTSTS strategy in desynchronizing 2,000 neuron E-I network in the presence of E-to-E and I-to-I synaptic connectivity. **(E)** Efficacy of the FTSTS strategy in desynchronizing 2,000 neuron E-I network where 25% of the population is inseparable and receives both the excitatory and inhibitory population input. The FTSTS pulse parameters for all the studies are *U*_*stim*_ = 100 mV, *T*_*stim*_ = 1 ms, and *T*_*neutral*_ = 10 ms.

In this paper, we developed and presented a novel stimulation strategy “Forced Temporal Spike-Time Stimulation” (FTSTS) for controlling synchronous activity of neurons in large spiking neural networks. Compared to other desynchronization strategies for large-scale spiking neural networks reported in the literature, our strategy focuses on controlling the average network synaptic weight by harnessing synaptic plasticity using a Hebbian-based spike-timing dependent plasticity (STDP) protocol that as a result controls the synchronization of neurons within the network. We presented a two neuron excitatory-inhibitory (E-I) network as an example to provide a mechanistic understanding of our approach. We later demonstrated the efficacy and robustness of the FTSTS strategy on large networks by varying the model parameters, synaptic connectivity and noisy inputs to the network. These results are also summarized in Figure 13 for clarity. While we only considered a LIF network, our method will lead to similar outcomes qualitatively for other neuronal models, since our approach is based on Hebbian activity-dependent plasticity. One of the prominent features of our FTSTS strategy is that it allows both synchronization and desynchronization of network activity by reversing the stimulation protocol (see Figures 3, 4), thus provides a complete control over the synchronization level of neural activity within a given network.

Our FTSTS strategy differs from existing stimulation strategies for desynchronizing spiking neural networks in many ways. Our strategy is based on harnessing the underlying synaptic plasticity compared to most of the desynchronization strategies reported in literature (Hauptmann et al., 2005; Popovych et al., 2005, 2017; Deuschl et al., 2006; Kiss et al., 2007; Nabi et al., 2013b; Mauroy et al., 2014; Wilson and Moehlis, 2014; Vlachos et al., 2016; Monga et al., 2018). Most of these strategies ignore the inherent synaptic plasticity among neurons in the network in designing the stimulation protocol for desynchronizing the network activity (One exception is “Coordinate Reset” (CR) (Tass, 2003a,b; Tass and Majtanik, 2006; Tass and Hauptmann, 2007; Pfister and Tass, 2010; Ebert et al., 2014; Zeitler and Tass, 2015). As a result, these strategies effectively desynchronize the network activity if the stimulation protocol is active. Once the stimulation protocol is turned off, the network resynchronizes rapidly because of the disappearance of the asynchronous regime in the absence of stimulation. Our strategy alleviates this problem, like CR, by explicitly incorporating and harnessing Hebbian-based STDP, which allows the network to stay in the asynchronous regime for a longer-time period after the stimulation is turned off (see Figure 3).

**Figure 14:**
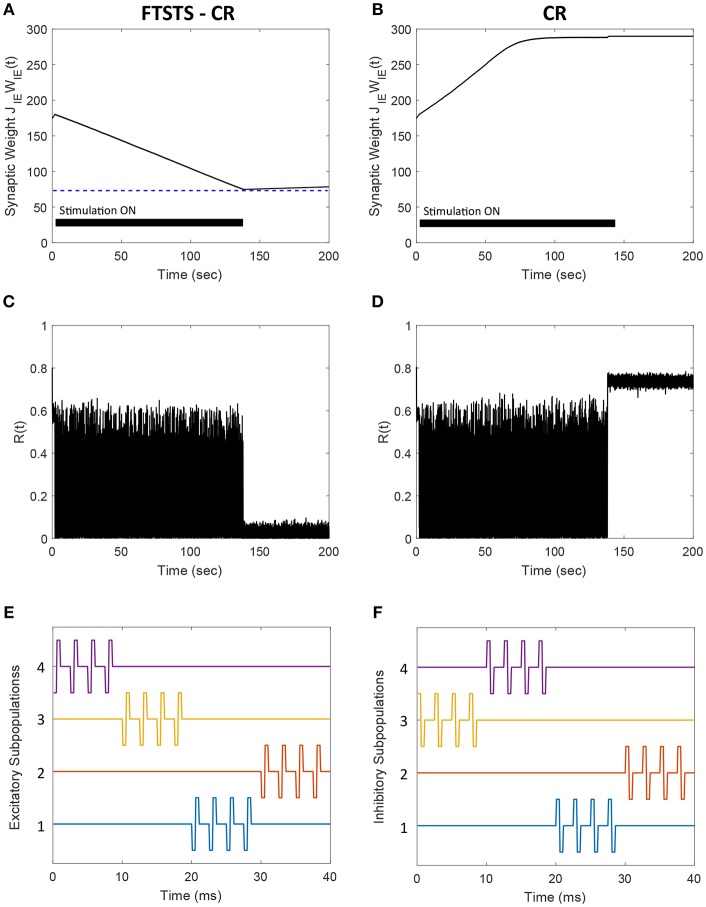
Comparison of the FTSTS-CR stimulation strategy with the coordinate-reset (CR) stimulation strategy on a network where LTP dominates LTD. **(A,C)** Show the changes in the average E-to-I synaptic weight of the network and the network synchrony level, respectively, for the FTSTS-CR stimulation strategy. **(B,D)** Show the changes in the average E-to-I synaptic weight of the network and the network synchrony level, respectively, for the CR stimulation strategy. **(E,F)** Show the FTSTS-CR stimulation pattern. The FTSTS-CR pulse parameters are *U*_*stim*_ = 200 mV, *T*_*stim*_ = 0.5 ms, and *T*_*neutral*_ = 3 ms. The STDP plasticity parameters are *a*_*LTD*_ = −1, *a*_*LTP*_ = 1.01,τ_*LTD*_ = 20 ms, and τ_*LTP*_ = 20 ms.

Almost all the stimulation strategies focus on desynchronizing the network activity by randomizing the firing patterns of neurons through direct stimulation. In comparison, our FTSTS strategy focuses on decreasing the average synaptic weight of the network by taking advantage of the Hebbian-based STDP protocol, which leads to the desynchronization of network activity. For example, the CR-based stimulation strategy desynchronizes the network activity by forcing different subpopulations of neurons to fire out of phase with each other, which resets the phase and desynchronizes the network (Tass, 2003a,b; Tass and Majtanik, 2006; Tass and Hauptmann, 2007; Pfister and Tass, 2010; Ebert et al., 2014; Zeitler and Tass, 2015). This generates an artificial asynchronous firing pattern that increases the basin of attraction of the asynchronous regime (i.e., lower synaptic weight stability point) (Pfister and Tass, 2010; Popovych and Tass, 2014). The underlying synaptic plasticity within the network then drives the average synaptic weight of the network toward the lower synaptic weight stability point (see Figures 10, 14 for comparison of our approach to the CR-based stimulation strategy).

Our developed framework can be incorporated into other desynchronization strategies, such as CR, to improve their efficacy. Figure 10 shows a comparison between the FTSTS-CR and CR performances in desynchronizing a E-I network consisting of 2,000 neurons with E-I synaptic plasticity (see section 3.8 for details of model parameters and specifics about the design of FTSTS-CR stimulation strategy). Since the FTSTS-CR stimulation strategy focused on decreasing the average synaptic weight of network, which as a result desynchronized the neural activity (see Figures 10B,D), this strategy outperformed the CR stimulation strategy shown in Figures 10A,C.

One of the limitations of the CR stimulation strategy is that the long-lasting effects occur only in networks where the long-term depression (LTD) dominates the long-term potentiation (LTP) of the synapses on average so that the network exhibits bistability (Pfister and Tass, 2010). It has been found that LTP dominates in specific aberrant neuronal pathways and brain regions such as the striatum indirect pathway underlying Parkinson's disease and hippocampus underlying epilepsy (Mathern et al., 1998; Johnston, 2004; Shen et al., 2008). In such brain networks, the CR and FTSTS-CR stimulation strategy would both fail to produce long-lasting desynchronization of the network activity but the FTSTS-CR will have a longer acute desynchronization effect compared to the CR protocol. To demonstrate this, we applied the CR stimulation strategy to an E-I network consisting of 2,000 neurons where LTP dominates LTD. As shown in Figure 14D, the network synchrony level is disrupted during the stimulation period of 140 s but increased to a synchrony level of *R*(*t*) = 0.7 after removal of the stimulus. Since CR stimulation, in this scenario, only induces acute desynchronization of the network and does not reduce the average synaptic weight, the average synaptic weight of the network remains in the synchronous regime the entire time, as shown in Figure 14B. As a result, the network resynchronized rapidly after the removal of the CR stimulus. We compare the desynchronization efficacy of the FTSTS-CR and CR approach in Figures 14A,B, respectively. During the period of FTSTS-CR stimulation, there is a decrease in the average synaptic weight (see Figure 14A). This results in a reduction in the synchrony level to *R*(*t*) = 0.05 when the FTSTS-CR stimulus is removed. While this desynchronization is transient due to the domination of LTP, the network remains desynchronized for a longer period of time compared to CR as shown in Figure 14C.

In this work, we have considered excitatory-inhibitory (E-I) networks with plastic E-to-I synapses. In general, our approach is applicable to other types of spiking neural networks such as purely excitatory or inhibitory networks as well as to networks with other plastic synapses such as E-to-E or I-to-I synapses. One of the limitations of our approach is that it assumes the same stimulus waveform is delivered to individual neurons within a subpopulation. Although we have demonstrated in simulation that our FTSTS strategy effectively desynchronizes the neuronal firings in a network even when the stimulation waveform parameters for individual neurons are drawn randomly from a given distribution (see Figure 6) and are contaminated with input designed for the opposite population (Figure 9), it is still able to utilize the relationship between pre and post firings to effectively harness the synaptic plasticity. Multi-laser optogenetics and recent development in optogenetics to excite or inhibit the same neuron using two different light wavelengths could potentially alleviate this limitation for experimental implementation of our strategy (Forli et al., 2018). Additionally, we assumed the majority of the excitatory and inhibitory neuron populations were spatially separate, which allows for the neuron populations to be separately stimulated. Two examples of spatially separate excitatory and inhibitory neuron populations are the striatum and cortex or the globus pallidus external segment (GPe) and subthalamic nucleus (STN) (Lanciego et al., 2012; Hegeman et al., 2016). The GPe-STN network has traditionally been targeted for DBS-HFS to treat PD and could be potential area to test this hypothesis. Although we have not optimized the FTSTR pulses to achieve a better performance or to make it more energy efficient, it is not difficult to formulate optimization problems that minimizes the average synaptic weight, network synchrony level, and applied stimulation energy simultaneously to achieve a better overall performance.

## Data Availability

All datasets generated for this study are included in the manuscript and/or the supplementary files.

## Author Contributions

GK and JS formulated the problem, discussed the results, and wrote the manuscript. JS developed the model, designed the FTSTS protocol, and ran the simulations.

### Conflict of Interest Statement

The authors declare that the research was conducted in the absence of any commercial or financial relationships that could be construed as a potential conflict of interest.

## References

[B1] Abbott L. F. Nelson S. B. (2000). Synaptic plasticity: taming the beast. Nat. Neurosci. 3:1178. 10.1038/81453 11127835

[B2] Bell C. C. Han V. Z. Sugawara Y. Grant K. (1997). Synaptic plasticity in a cerebellum-like structure depends on temporal order. Nature 387:278. 9153391 10.1038/387278a0

[B3] Benabid A. L. Chabardes S. Mitrofanis J. Pollak P. (2009). Deep brain stimulation of the subthalamic nucleus for the treatment of Parkinson's disease. Lancet Neurol. 8, 67–81. 10.1016/S1474-4422(08)70291-6 19081516

[B4] Brunel N. Hansel D. (2006). How noise affects the synchronization properties of recurrent networks of inhibitory neurons. Neural Comput. 18, 1066–1110. 10.1162/neco.2006.18.5.1066 16595058

[B5] Daido H. (1992). Order function and macroscopic mutual entrainment in uniformly coupled limit-cycle oscillators. Progress Theoret. Phys. 88, 1213–1218.

[B6] Deuschl G. Schade-Brittinger C. Krack P. Volkmann J. Schäfer H. Bötzel K.. (2006). A randomized trial of deep-brain stimulation for Parkinson's disease. N. Eng. J. Med. 355, 896–908. 10.1056/NEJMoa060281 16943402

[B7] Duncan J. S. Sander J. W. Sisodiya S. M. Walker M. C. (2006). Adult epilepsy. Lancet 367, 1087–1100. 10.1016/S0140-6736(06)68477-8 16581409

[B8] Ebert M. Hauptmann C. Tass P. A. (2014). Coordinated Reset stimulation in a large-scale model of the STN-Gpe circuit. Front. Comput. Neurosci. 8:154. 10.3389/fncom.2014.00154 25505882 PMC4245901

[B9] Forli A. Vecchia D. Binini N. Succol F. Bovetti S. Moretti C.. (2018). Two-photon bidirectional control and imaging of neuronal excitability with high spatial resolution *in vivo*. Cell Rep. 22, 3087–3098. 10.1016/j.celrep.2018.02.063 29539433 PMC5863087

[B10] Hammond C. Bergman H. Brown P. (2007). Pathological synchronization in Parkinson's disease: networks, models and treatments. Trends Neurosci. 30, 357–364. 10.1016/j.tins.2007.05.004 17532060

[B11] Hauptmann C. Popovych O. Tass P. A. (2005). Delayed feedback control of synchronization in locally coupled neuronal networks. Neurocomputing 65, 759–767. 10.1016/j.neucom.2004.10.072

[B12] Hauptmann C. Tass P. (2009). Cumulative and after-effects of short and weak coordinated reset stimulation: a modeling study. J. Neural Eng. 6:016004. 10.1088/1741-2560/6/1/016004 19141875

[B13] Hebb D. O. (1949). The Organization of Behavior. New York, NY: Wiley.

[B14] Hegeman D. J. Hong E. S. Hernández V. M. Chan C. S. (2016). The external globus pallidus: progress and perspectives. Eur. J. Neurosci. 43, 1239–1265. 10.1111/ejn.13196 26841063 PMC4874844

[B15] Johnston M. V. (2004). Clinical disorders of brain plasticity. Brain Dev. 26, 73–80. 10.1016/S0387-7604(03)00102-5 15036425

[B16] Kiss I. Z. Rusin C. G. Kori H. Hudson J. L. (2007). Engineering complex dynamical structures: sequential patterns and desynchronization. Science 316, 1886–1889. 10.1126/science.1140858 17525302

[B17] Klinger N. V. Mittal S. (2016). Clinical efficacy of deep brain stimulation for the treatment of medically refractory epilepsy. Clin. Neurol. Neurosurg. 140, 11–25. 10.1016/j.clineuro.2015.11.009 26615464

[B18] Kuramoto Y. (1984). Chemical Oscillations, Waves, and Turbulence. Berlin: Springer.

[B19] Lanciego J. L. Luquin N. Obeso J. A. (2012). Functional neuroanatomy of the basal ganglia. Cold Spring Harbor Perspect. Med. 2:a009621. 10.1101/cshperspect.a009621 23071379 PMC3543080

[B20] Luz Y. Shamir M. (2012). Balancing feed-forward excitation and inhibition via Hebbian inhibitory synaptic plasticity. PLoS Comput. Biol. 8:e1002334. 10.1371/journal.pcbi.1002334 22291583 PMC3266879

[B21] Mathern G. W. Pretorius J. K. Leite J. P. Kornblum H. I. Mendoza D. Lozada A.. (1998). Hippocampal AMPA and NMDA mRNA levels and subunit immunoreactivity in human temporal lobe epilepsy patients and a rodent model of chronic mesial limbic epilepsy. Epilepsy Res. 32, 154–171. 9761317 10.1016/s0920-1211(98)00048-5

[B22] Mauroy A. Rhoads B. Moehlis J. Mezic I. (2014). Global isochrons and phase sensitivity of bursting neurons. SIAM J. Appl. Dynam. Syst. 13, 306–338. 10.1137/130931151

[B23] Monga B. Froyland G. Moehlis J. (2018). Synchronizing and desynchronizing neural populations through phase distribution control, in 2018 Annual American Control Conference (ACC) (Milwaukee, WI: IEEE), 2808–2813.

[B24] Nabi A. Mirzadeh M. Gibou F. Moehlis J. (2013a). Minimum energy desynchronizing control for coupled neurons. J. Comput. Neurosci. 34, 259–271. 10.1007/s10827-012-0419-3 22903565

[B25] Nabi A. Stigen T. Moehlis J. Netoff T. (2013b). Minimum energy control for *in vitro* neurons. J. Neural Eng. 10:036005. 10.1088/1741-2560/10/3/036005 23574761

[B26] Pfister J.-P. Tass P. A. (2010). STDP in oscillatory recurrent networks: theoretical conditions for desynchronization and applications to deep brain stimulation. Front. Comput. Neurosci. 4:22. 10.3389/fncom.2010.00022 20802859 PMC2928668

[B27] Popovych O. V. Hauptmann C. Tass P. A. (2005). Effective desynchronization by nonlinear delayed feedback. Phys. Rev. Lett. 94:164102. 10.1103/PhysRevLett.94.164102 15904229

[B28] Popovych O. V. Lysyansky B. Rosenblum M. Pikovsky A. Tass P. A. (2017). Pulsatile desynchronizing delayed feedback for closed-loop deep brain stimulation. PloS ONE 12:e0173363. 10.1371/journal.pone.0173363 28273176 PMC5342235

[B29] Popovych O. V. Tass P. A. (2014). Control of abnormal synchronization in neurological disorders. Front. Neurol. 5:268. 10.3389/fneur.2014.00268 25566174 PMC4267271

[B30] Postuma R. B. Berg D. Stern M. Poewe W. Olanow C. W. Oertel W.. (2015). MDS clinical diagnostic criteria for Parkinson's disease. Movement Disord. 30, 1591–1601. 10.1002/mds.26424 26474316

[B31] Shen W. Flajolet M. Greengard P. Surmeier D. J. (2008). Dichotomous dopaminergic control of striatal synaptic plasticity. Science 321, 848–851. 10.1126/science.1160575 18687967 PMC2833421

[B32] Singh A. (2018). Oscillatory activity in the cortico-basal ganglia-thalamic neural circuits in Parkinson's disease. Eur. J. Neurosci. 48, 2869–2878. 10.1111/ejn.13853 29381817

[B33] Song S. Miller K. D. Abbott L. F. (2000). Competitive hebbian learning through spike-timing-dependent synaptic plasticity. Nat. Neurosci. 3:919. 10.1038/78829 10966623

[B34] Tass P. A. (2003a). Desynchronization by means of a coordinated reset of neural sub-populations: a novel technique for demand-controlled deep brain stimulation. Progress Theoret. Phys. Suppl. 150, 281–296. 10.1143/PTPS.150.281

[B35] Tass P. A. (2003b). A model of desynchronizing deep brain stimulation with a demand-controlled coordinated reset of neural subpopulations. Biol. Cybernet. 89, 81–88. 10.1007/s00422-003-0425-7 12905037

[B36] Tass P. A. (2007). Phase Resetting in Medicine and Biology: Stochastic Modelling and Data Analysis. Berlin: Springer Science & Business Media.

[B37] Tass P. A. Hauptmann C. (2007). Therapeutic modulation of synaptic connectivity with desynchronizing brain stimulation. Int. J. Psychophysiol. 64, 53–61. 10.1016/j.ijpsycho.2006.07.013 16997408

[B38] Tass P. A. Hauptmann C. (2009). Anti-kindling achieved by stimulation targeting slow synaptic dynamics. Restorat. Neurol. Neurosci. 27, 589–609. 10.3233/RNN-2009-0484 20042784

[B39] Tass P. A. Majtanik M. (2006). Long-term anti-kindling effects of desynchronizing brain stimulation: a theoretical study. Biol. Cybernet. 94, 58–66. 10.1007/s00422-005-0028-6 16284784

[B40] Temperli P. Ghika J. Villemure J.-G. Burkhard P. R. Bogousslavsky J. Vingerhoets F. J. (2003). How do Parkinsonian signs return after discontinuation of subthalamic DBS? Neurology 60, 78–81. 10.1212/WNL.60.1.78 12525722

[B41] Vlachos I. Deniz T. Aertsen A. Kumar A. (2016). Recovery of dynamics and function in spiking neural networks with closed-loop control. PLoS Comput. Biol. 12:e1004720. 10.1371/journal.pcbi.1004720 26829673 PMC4734620

[B42] Wilson D. Moehlis J. (2014). Optimal chaotic desynchronization for neural populations. SIAM J. Appl. Dynam. Syst. 13:276 10.1137/120901702 PMC4159599 24899243

[B43] Zeitler M. Tass P. A. (2015). Augmented brain function by coordinated reset stimulation with slowly varying sequences. Front. Syst. Neurosci. 9:49. 10.3389/fnsys.2015.00049 25873867 PMC4379899

